# The Role of Genetic Explanations of Substance Use Disorders in Shaping Stigma in the General Public

**DOI:** 10.1159/ear/acrag007

**Published:** 2026-09-18

**Authors:** Anaïs B. Thijssen, Rada R. Veeneman, Anil P.S. Ori, Karin J.H. Verweij

**Affiliations:** a Department of Psychiatry, Amsterdam UMC, University of Amsterdam, Amsterdam, The Netherlands; b Amsterdam Public Health, Amsterdam, The Netherlands; c Amsterdam Neuroscience, Amsterdam, The Netherlands; d Amsterdam Cardiovasculair Sciences, Amsterdam, The Netherlands

**Keywords:** Addiction, Genetics, Literature review, Stigma

## Abstract

**Background:**

Substance use disorders (SUDs) are among the most highly stigmatized mental health conditions, with stigma acting as a key barrier to treatment engagement and recovery. SUDs are especially stigmatized as they are more likely to be viewed as voluntary conditions, compared to other mental health illnesses. As genetic research increasingly illuminates the biological underpinnings of addiction, it is important to know whether this understanding reduces or reinforces stigma in the general public.

**Summary:**

We conducted a review to identify studies examining the relationship between genetic explanations of SUDs and stigmatizing attitudes. Across both correlational and experimental studies, genetic attributions consistently reduced personal blame toward individuals with SUDs. This finding is consistent with attribution theory, which states that individuals will be blamed less if their condition is viewed as outside their control. Effects on other dimensions of stigma, including perceptions of dangerousness, fear, and desire for social distance, were inconsistent across studies. Studies were limited by the use of deterministic, binary genetic framings that do not accurately reflect the complex interplay of genetic and environmental factors underlying addiction risk.

**Key Messages:**

Genetic attributions consistently reduced personal blame toward individuals with SUDs, while associations with other stigma-related attitudes were less clear. Within the disease-model framework, reduced blame is interpreted as a reduction in stigma-related attitudes, although frameworks emphasizing individual agency may interpret this finding differently. Future research should adopt scientifically accurate gene-environment frameworks and reevaluate the effect on stigma, attend to the needs of individuals with lived experience of addiction, and examine how changes in interpersonal stigma interact with structural stigma. Careful communication of genetic findings is essential to avoid reinforcing essentialist views of addiction.

## Introduction

Substance use disorders (SUDs) are one of the most highly stigmatized mental health conditions [1]. Stigma is a process in which a person or group is devalued, discredited, and/or discriminated against because of a particular attribute, condition, or identity that is socially constructed as undesirable [2]. Stigma exists at the structural level (e.g., within policies and institutions), at the interpersonal level (e.g., stigma in general public), and at the individual level (e.g., self-stigma) [3]. At the interpersonal level, stigma refers to interactions between stigmatized and non-stigmatized individuals, which can encompass stereotypes (beliefs about certain individuals), prejudice (negative emotional reactions), and discrimination (negative behavioral responses) [4, 5]. Being stigmatized causes shame and social isolation, which leads to resistance to seek help and is associated with reduced recovery [4]. Stigma can therefore exacerbate the already high mortality and morbidity associated with SUDs.

Compared to schizophrenia and depression, SUDs are seen more as a chosen affliction and less as a mental illness, and individuals living with an SUD are regarded as more responsible for their condition [1]. This belief in culpability in turn leads to more social rejection and more discrimination of people living with SUDs than is the case for people living with other types of mental illnesses [5]. Policies associated with the war on drugs have often emphasized criminalization and punishment rather than treatment, reflecting an emphasis on individual responsibility for substance use [6]. Within a disease-model framework, such policies may be understood as a form of structural stigma because they treat addiction primarily as voluntary misconduct rather than as a health condition requiring care. The disease model of addiction builds on research showing that repeated substance use causes neurobiological changes in brain circuits governing reward, motivation, and self-control, rendering addictive behavior compulsive and substantially outside of voluntary control [7]. This model of addiction has become the dominant view of addiction, although many also regard it as an oversimplification [8, 9], or note that it overemphasizes biological mechanisms and does not adequately account for the role of personal choice and agency [10]. Some have even argued that stigma might serve a functional role in the case of SUDs, by acting as a discouraging pressure, deterring the use of socially disapproved substances as well as excessive or problematic patterns of substance use [11], while others argue that the stigma concept is misapplied to addiction altogether, as societal disapproval of voluntary harmful behavior does not constitute unjust devaluation [12]. These views assume that substance use is a choice and that people with an addiction can be held personally responsible for their substance use. Even within a disease framework, however, responsibility and blame may be seen as separate concepts [13], and societal disapproval of substance use should be distinguished from the stigmatization of people with SUDs. However, societal disapproval may deter voluntary aspects of substance use or encourage cessation [4]. Moreover, although biological and other factors outside personal control do not necessarily exclude a role for personal agency, their contribution to SUD risk complicates attributions of individual blame and may limit the extent to which stigmatization can effectively deter substance use [4].

Within the disease-model framework, understanding the biological mechanisms of addiction may be relevant to perceptions of personal culpability and, consequently, stigma, even though biological involvement does not in itself negate personal responsibility. Over the past decades, research investigating the genetic and biological underpinnings of addiction showed that a complex interplay of genetic and environmental factors contributes to one’s vulnerability for developing an SUD. Evidence that genetic factors are associated with individual differences in addiction risk may affect how society views these conditions, by reducing perceptions of personal responsibility for them [14]. Alternatively, it might contribute to beliefs that individuals suffering from SUDs are somehow different [15].

In this review, we explore the impact of genetic explanations of SUDs in shaping stigma in the general public. We adopt the disease model of addiction as its conceptual framework, consequently, reductions in blame, perceived responsibility, social distance, and negative affect are interpreted as reductions in stigma-related attitudes.

### Genetics of SUDs

Individuals differ in their vulnerability for developing an addiction. Twin studies have estimated that the heritability of SUDs is in the range of 50–70% [16–18]; meaning that 50–70% of individual differences in the propensity to develop a SUD can be explained by genetic differences between people, while the remaining 30–50% arises from environmental or other nongenetic factors. Heritability is a population-level statistic describing the degree to which genetic differences between people account for variation in a trait across a population and cannot be interpreted as a genetic explanation for any particular individual's substance use. Nowadays, large-scale genome-wide association studies, using DNA data from hundreds of thousands of participants, are helping to pinpoint the specific genetic variants involved and to shed light on the genetic architecture and biological mechanisms that contribute to addiction [19–21]. Importantly, the fact that a trait is heritable does not mean that people’s outcomes are fixed by their genetics. Whether or not someone develops an addiction depends on a complex interplay between genetic vulnerability and a range of environmental factors, such as drug availability, exposure to early life adversity, social-economic status, and peer use [22]. In addition, not all genetic associations are necessarily related to biological processes, as they can also reflect environmental effects, due to for instance gene-environment correlations with social-economic status, or systematic biases [23, 24].

### Perceiving Addiction as Genetic: Countering or Reinforcing Stigma?

We reviewed the literature to investigate whether perceiving addiction as genetic counters or reinforces stigma. We used a search string in Scopus that encompassed different addiction, stigma, and genetic keywords (Table 1). This search resulted in 76 articles, which were subsequently screened based on title and abstract. We included 11 studies that focused on our research question. These studies were carried out between 2014 and 2024, differed in sample size from N = 252 to over 4,000 individuals, with the majority focusing on alcohol use disorder, conducted in either the USA or Germany (Table 2). We note that some of these studies were more detailed than we describe them here, as we focused solely on our research question.

**Table 1.** Search string used in Scopus

Addiction	TITLE-ABS(addiction) OR TITLE-ABS (alcohol use disorder*) OR TITLE-ABS (alcohol dependen*) OR TITLE-ABS (alcohol abus*) OR TITLE-ABS (substance use disorder*) OR TITLE-ABS (substance dependen*) OR TITLE-ABS (substance abuse*) OR TITLE-ABS (opioid use disorder*) OR TITLE-ABS (opioid dependen*) OR TITLE-ABS (opioid abus*) OR TITLE-ABS (cannabis use disorder*) OR TITLE-ABS (cannabis dependen*) OR TITLE-ABS (cannabis abus*) OR TITLE-ABS (heroin use disorder*) OR TITLE-ABS (heroin dependen*) OR TITLE-ABS (heroin abus*) OR TITLE-ABS (cocaine use disorder*) OR TITLE-ABS (cocaine dependen*) OR TITLE-ABS (cocaine abus*)
Genetic explanation	AND (TITLE-ABS (genet*) OR TITLE-ABS (biogenet*))
Stigma	AND (TITLE-ABS (Social distanc*) OR TITLE-ABS (blam*) OR TITLE-ABS (stigma*))
General	AND (LIMIT-TO (DOCTYPE, “ar”)) AND (LIMIT-TO (LANGUAGE, “English”))

**Table 2.** Summary of the included studies

Study	Study design	Type of SUD	*N*	Genetic explanation	Stigma measures	Effect of genetic attributions
Pescosolido et al. [25] (2021)	Observational; vignette depicting someone with an addiction; no correlation analysis was done	AUD	1,438 (1996), 1,520 (2006), 1,171 (2018)	Participants indicated whether they thought the addiction was caused by genetics	Dangerousness: the likelihood that the person would do something violent. Social distance: items measuring willingness to be socially involved. Bad character: addiction due to bad character	The belief that it was genetic: increased (from 58% to 69%, *F =* 3.5, *p* = 0.03); dangerousness: no change; social distance: no change; bad character: increased (from 49% to 65%, *F* = 7.8, *p* = 0.00)
Burnette et al. [26] (2025) – part 1	Correlational; no vignette used	Substance addiction	294	Participants indicated the degree to which the addiction is due to genetics, the environment, or personal choice, with scores adding to 100%. Scores for genetics were used in analyses	Prejudice: how warm/cold they felt toward a person with an addiction. Stereotypes: whether they endorsed negative stereotypes. Discrimination: whether they endorsed discriminatory items	Prejudice: decreased (*r* = −0.29, *p* ≤ 0.001); stereotypes: decreased (*r* = −0.34, *p* ≤ 0.001); discrimination: decreased (*r* = −0.32, *p* < 0.001)
Burnette et al. [26] (2025) – part 2	Correlational; no vignette used	Substance addiction	283	Participants indicated how strongly they thought the addiction is due to genetics	Blame: items on whether people with addictions are to blame for their addiction. Stereotypes: whether they endorsed negative stereotypes. Discrimination: whether they endorsed discriminatory items	Blame: reduced (*r* = −0.36, *p* = < 0.001); stereotypes: no effect; discrimination: reduced (*r* = −0.22, *p* ≤ 0.001)
Schomerus et al. [27] (2014)	Correlational; vignette study	AUD	1,126	Participants indicated how strongly they thought the addiction was due to biogenetic causes (a mix of chemical imbalance in the brain, brain disease, and heredity)	Social acceptance: items measuring willingness to be socially involved. Blame: the person is responsible for the addiction. Differentness: the person is different from other people. Dangerousness: the person is dangerous	Blame: reduced (coef = −0.09, *p* < 0.05); differentness: increased (coef = 0.20, *p < 0.05*); dangerousness: increased (coef 0.21, *p* < 0.05); social acceptance: increased (coef = 0.17, *p* = 0.17)
Speerforck et al. [28] (2014)	Correlational; vignette study	AUD	1,141	Participants indicated how strongly they thought addiction was due to heredity	Social distance: items measuring willingness to be socially involved. Fear and prosocial reactions: based on principal component analysis of questionnaire on emotional reactions	Social distance: no effect; fear: increased (Beta = 0.12, *p* < 0.001); prosocial reactions: no effect
Jacobi et al. [29] (2022)	Correlational; vignette study	SUD	∼760	Participants indicated how strongly they thought the addiction was due to a genetic or inherited problem	Social distance: items measuring willingness to be socially involved	Social distance: no effect
Siddiqui & Rutherford, [30] (2023)	Experimental	OUD, methamphetamine	252	Participants read a news article indicating that scientists discovered the genetic basis of addiction or that there definitely was no genetic basis	Combined scale with items measuring social stigma and structural stigma	No effect on the combined scale
Lebowitz & Appelbaum, [31] (2017)	Experimental; vignette study	AUD, GD	403	Participants were told that the addiction of the vignette character was either caused by genetics or was definitely not caused by genetics	Blame: how much the participants blamed the vignette character for their addiction	Blame: decreased (*F*(1,399) = 49.5, *p* < 0.001, *d* = 0.71)
Sattler et al. [32] (2017)	Experimental; vignette study	AUD, cocaine	2,795	The vignette character either had or did not have a genetic predisposition for addiction	Dangerousness: whether the participant thought the vignette character was dangerous. Blame: whether the condition is the vignette character’s own fault. Fear: whether the participant would feel scared of the vignette character. Avoidance: whether the participant would try to stay away from the vignette character	Dangerousness: no change; blame: decreased (OR = 0.74, *p* < 0.001); fear: no effect; avoidance: no effect
Elliot et al. [33] (2024)	Experimental; vignette study	AUD, OUD	∼321	The condition of the vignette character was either said to be likely genetic, likely genetic and environmental, likely environmental, or no explanation was given	Social distance: items measuring willingness to be socially involved	Social distance: increased for the genetic explanation compared to when no explanation was given (*b* = 0.20, *β* = 0.09; *p* = 0.002)
Strzelecki & Waldron, [34] (2021)	Experimental; vignette study	AUD	449	The vignette character was either said to have a genetic predisposition for addiction or no information was given	Social distance: items measuring willingness to be socially involved. Blame: items measuring blame	Social distance: decreased *F*(1, 441) = 5.51, *p* = 0.02, $\eta_{\rho }^{2}$ = 0.01); blame decreased (*F*(1,441) = 8.93, *p* = 0.003, $\eta_{\rho }^{2}$ = 0.02)
Lebowitz et al. [35] (2024)	Experimental; vignette study	AUD, GD	385	The vignette character was either said to have a genetic predisposition for addiction or to not have a genetic predisposition	Blame: the degree to which the vignette character is to blame for the addiction	Blame: decreased (related to personal attributions, not to the experimental manipulation; partial correlation = −0.17, *p* = 0.001)

AUD, alcohol use disorder; SUD, substance use disorder, OUD, opioid use disorder, GD, gambling disorder.

Five observational studies have examined the influence of perceiving SUDs as genetic on stigma, by administering questionnaires that measure the extent to which the public attributes addiction to genetic causes and asks about their views of individuals with addiction. Pescosolido and colleagues [25] surveyed nationally representative samples of the USA in 1996, 2006, and 2018, regarding their attitudes toward individuals with alcohol use disorder and their beliefs about its causes. Over this period, endorsement of genetic explanations increased, social acceptance and perceived dangerousness remained unchanged, whereas endorsement of bad character as a cause of alcohol dependence (a stigmatizing stereotype) increased. However, because the study examined population trends over time rather than direct associations, it cannot establish whether the increased endorsement of genetic explanations contributed to these patterns. Burnette and colleagues [26] directly investigated whether genetic beliefs were associated with reduced stigma and found that individuals who believe that substance addiction is due to genetics were less likely to have stigmatizing views, as they were less likely to attribute addiction to bad character or to a person’s own fault. An increased belief that addiction is due to genetics was furthermore associated with less prejudice and stereotyping and less desire for social distance (not wanting to be socially involved). These findings were largely confirmed in an independent replication sample within the same study, with the exception of stereotype endorsement, which did not reach significance. A similar finding was presented in another study [27], which showed that an increased belief that alcohol use disorder symptoms were due to biogenetic causes was associated with a decrease in blame and social distance but also with an increase in perceptions of dangerousness and differentness. The study revealed that the effect of biogenetic causal beliefs on social distance was mediated by blame, differentness, and dangerousness. However, the biogenetic scale was made up of “chemical imbalance in the brain,” “brain disease,” and “heredity,” and the decrease in social distance was no longer significant when only assessing heredity as a cause for alcohol use disorder in a second study [28]. A positive association of biogenetic causal explanations with fear was found for all three types of biogenetic explanations, while only heredity showed no increase in prosocial reactions. Similarly, in faith communities in the USA, believing that addiction symptoms of a vignette character were due to a “genetic or inherited problem” was unrelated to a desire for social distance [29].

Six studies experimentally manipulated the belief that addiction is due to genetics and examined the impact on stigmatizing attitudes. In one of these studies [30], half of the participants read an article describing that a scientific study had found the genetic basis of opioid or methamphetamine addiction, while the other half read that there was definitely no genetic basis. The participants subsequently completed a questionnaire asking about social (whether they wanted to be socially involved) and structural (whether they thought the government should spend money on treating addiction) stigma. Only the effect on the combined scale of social and structural stigma was reported, revealing no association with the genetic manipulation. However, the articles likely did not have the desired effect, as they were not associated with the manipulation check on belief in biological essentialism (i.e., the belief that addiction is caused by genetics). Given the small sample, the use of the combined scale, and the failed manipulation check, it is difficult to draw conclusions on whether a genetic explanation impacts stigma.

In another study, participants who read about a person who was addicted to alcohol or gambling viewed the vignette character as less blameworthy when the addiction was said to be caused by their genes compared to when it was said to not be caused by genetics [31]. A similar vignette study investigating alcohol and cocaine addiction in a larger sample [32] also showed that learning that someone’s addiction was due to a genetic predisposition reduced blame compared to learning that the vignette character was said to have no genetic predisposition. However, the genetic explanation had no effect on feelings of fear, avoidance, or perception of dangerousness. A later study found that providing a genetic explanation for alcohol or opioid use disorder increased the desire for social distance compared to when no explanation was provided, which was not the case when an environmental or a combination of an environmental and genetic explanation was provided [33]. In contrast, in a study conducted a few years earlier [34], desire for social distance was reduced when the vignette character had a genetic predisposition for addiction compared to when there was no genetic predisposition, while again confirming that the genetic explanation leads to reduced blame. A reduction in blame was also found for professionals that treat individuals with addictions [35]. Interestingly, in this study, only the personal belief the professionals had about whether gambling or alcohol use disorder was caused by genetics showed this effect, while the experimental manipulation did not have an effect, most likely because the professionals did not change their beliefs that much because of the manipulation.

Across both correlational and experimental studies, a clear pattern emerges for one aspect of stigma: Genetic attributions consistently reduced personal blame toward individuals with addiction, while effects on other forms of stigma were less consistent. In light of these findings, we next examine a likely mechanism (attribution theory) that may account for the effects of genetic explanations on stigma, the impact of genetics explanations on individuals with SUDs, and the limitations of the existing studies.

### How Genetic Explanations Might Influence Stigma

Attribution theory proposes that individuals are blamed for their condition when they are perceived as personally responsible for its occurrence [36, 37]. Consistent with this framework, attributing SUDs to genetic causes is associated with reduced blame, as observed above. For other mental health conditions, genetic framing was associated with a similar decrease in blame, although higher perceptions of dangerousness and prognostic pessimism (the belief that the mental health condition cannot be overcome) were also observed [38, 39]. This pattern could be explained by genetic essentialism, which is the belief that genetic causes for a condition mean that people have no control over their actions or behaviors and the undesired outcome is inescapable, thereby increasing perceptions of dangerousness. However, such essentialist assumptions are scientifically unfounded for complex traits such as addiction [14, 40]. Attribution theory and genetic essentialism together help explain why genetic information produces mixed and contradictory effects in relation to addiction, reducing some aspects of stigma while amplifying others.

### The Impact of Learning about a Genetic Predisposition

Blame reduction toward individuals with SUDs is a beneficial consequence of emphasizing the genetic contributions to these conditions. However, it is also important to consider how such genetic explanations may affect individuals suffering from an addiction themselves. A potential unintended consequence is that people may perceive their addiction as immutable or predetermined, reducing their sense of agency for recovery. A qualitative study examining patients’ perspectives found substantial variability in how patients interpret genetic predisposition to SUDs [41]. Approximately one-third of the participants reported that such information would reduce guilt, facilitate acceptance of their condition, or encourage treatment engagement by countering the belief that they are “bad people who cannot change”. In contrast, two-thirds felt that genetic risk information would not enhance their motivation for recovery, emphasizing that sustained abstinence requires effort regardless of the cause of their addiction. A minority expressed concerns that genetic explanations might foster fatalistic beliefs, making recovery feel less attainable. In terms of perceived public attitudes, most participants believed that genetic explanations would reduce stigma by shifting attributions away from personal blame and toward greater compassion, which is in line with the findings from the studies above. Dar-Nimrod and colleagues [42] experimentally informed individuals that they either did or did not carry a genetic susceptibility to alcoholism (which in reality was not measured). Learning about having a genetic susceptibility reduced individuals’ perceived control over their drinking and increased the likelihood of enrolling in a responsible drinking workshop. However, learning about one’s genetic status did not affect the amount of alcohol the individuals planned to consume over the following month. This suggests that genetic feedback can simultaneously elicit feelings of reduced agency while also motivating engagement with supportive interventions.

### Challenges of Providing Genetic Explanations to Investigate Their Effects on Stigma

Decades of research from epidemiological, family-based, and molecular genetic studies have firmly established that addiction risk is shaped by a multitude of factors, including genetics and the environment and their interaction. The correlational studies reviewed above generally reflect this scientific consensus by asking participants to evaluate the degree to which multiple causal factors, including genetic and environmental influences, contribute to addiction. In contrast, nearly all experimental studies (with the exception of Elliot et al. [33], 2024, which included a condition where genes and environment both contributed to the onset) presented participants with a binary message that the addiction was either genetically caused or not genetically caused. This framing reinforces an artificial dichotomy and promotes deterministic interpretations, wrongly implying that genetic influences operate in isolation and that this predisposition inevitably will lead to the disorder. Such simplification is both scientifically inaccurate and limits the interpretability of these findings, as it does not reveal whether an accurate representation of the role of genetics in addictions reduces stigma. It furthermore reinforces public perceptions of genetic essentialism. Importantly, the potential increase in perceptions of dangerousness in response to genetic explanations may be mitigated when a more scientifically accurate gene-environment model is presented. However, this may also attenuate the blame-reduction effect observed in studies using deterministic genetic framings. By explicitly acknowledging that both genetic and environmental influences are involved in developing an addiction, the belief in genetic essentialism, i.e., that genetic causes render behaviors fixed and uncontrollable, might be combated. Furthermore, vignette studies may elicit less intense emotional reactions than real-life encounters (limiting ecological validity), and stigma-related questions may be susceptible to socially desirable responding (introducing response bias). In addition, the stigma-related questions did not encompass the full spectrum of stigma. Another important limitation is that these studies were conducted in the USA and Germany, which are both Western societies. Different cultural contexts influence how individuals with addictions are viewed and how genetic explanations would relate to stigma. In addition, the intersectionality of stigma is not addressed in these studies, which could be relevant, as some groups with addictions might be more stigmatized than others (e.g., difference between genders, racial-ethnic groups, and socioeconomic classes). Finally, it is important to note that our interpretations rely on adopting the disease model of addiction, which holds that SUDs are substantially outside an individual’s voluntary control. Under this model, negative public attitudes toward individuals with SUD constitute stigma and their reduction is desirable. Although the disease model is the dominant framework in stigma and SUD research, its validity remains debated, and our conclusions should be interpreted with this assumption in mind.

## Conclusion

Genetic framing of SUDs can reduce blame for individuals living with these conditions by lessening views of personal responsibility for its onset. Within the disease-model framework adopted in this review, these changes may be interpreted as reductions in certain stigma-related attitudes. However, this interpretation is not theoretically or normatively neutral: frameworks that place greater emphasis on individual agency may interpret reduced responsibility attribution differently. Whether other stigma-related attitudes (such as perceptions of dangerousness or desire for social distance) are also influenced by genetic framing and whether these associations are different for addiction than for other mental health conditions remain unclear, as there is currently only limited research investigating these questions. Importantly, future research should adopt a scientifically accurate and nondeterministic framework of the interplay of genetics and the environment to better understand how accurate genetic information influences stigma of addiction. Researchers should implement safeguards to avoid reinforcing essentialist and reductionist views of the role of genetics in addiction. Furthermore, attention should be paid to what this information elicits in patients and whether and under what circumstances it may benefit or harm their recovery. Finally, it will be important to understand how changes at the level of (inter)personal stigma interact with stigma at the structural level, and vice versa, and how interventions to reduce stigma in relation to SUDs can be best achieved.

## Conflict of Interest Statement

K.J.H.V. was a member of the journal’s Editorial Board at the time of submission. The remaining authors have no conflicts of interest to declare.

## Funding Sources

A.B.T. is supported by a PhD scholarship from Amsterdam University Medical Center. K.J.H.V. and A.P.S.O. are supported by the Foundation Volksbond Rotterdam. The funders had no role in study design, data collection and analysis, decision to publish, or preparation of the manuscript.

## Author Contributions

R.R.V. and K.J.H.V.: conceptualization. A.B.T. and R.R.V.: investigation, writing – original draft. K.J.H.V. and A.P.S.O.: writing – review and editing.
